# Targeting Dendritic Cells as a Good Alternative to Combat *Leishmania* spp.

**DOI:** 10.3389/fimmu.2014.00604

**Published:** 2014-11-26

**Authors:** Rafael Freitas-Silva, Maria Carolina Accioly Brelaz-de-Castro, Antônio Mauro Rezende, Valéria Rêgo Pereira

**Affiliations:** ^1^Department of Natural Sciences, University of Pernambuco, Garanhuns, Brazil; ^2^Department of Immunology, Aggeu Magalhães Research Center, Oswaldo Cruz Foundation, Recife, Brazil; ^3^Department of Microbiology, Aggeu Magalhães Research Center, Oswaldo Cruz Foundation, Recife, Brazil

**Keywords:** dendritic cells, vaccine, neglected diseases, leishmaniasis

## Leishmaniasis: Global Burden, Clinical Forms, and Current Strategies of Control

Leishmaniasis is an important group of neglected diseases caused by more than 20 spp. of protozoan from the genus *Leishmania*. It is transmitted by sandfly bite (1), and impacts populations by inducing disfiguration, loss of productivity, and a burden estimated at 2,357.000 disability-adjusted life years (DALY) (2). Ninety-eight countries have reported cases of leishmaniasis, and over 350 million people are living at risk, with 0.2–0.4 and 0.7–1.2 million cases of VL and CL annually, respectively (3). Three main clinical forms are known: visceral (VL, more lethal, e.g., *L. donovani* and *L. infantum*), cutaneous (CL, more common, e.g., *L. major*), and mucocutaneous (MCL). Strategies to limit these diseases are controlling the vectors and chemotherapy of affected individuals, but these approaches have a high cost and led to resistant parasites and vectors (4). Thus, there is an urgent need of vaccines and more effective therapies for leishmaniasis, otherwise the number of cases and resistant strains will probably continue to rise. Since they have a key capacity to initiate and maintain an immune response, dendritic cells (DCs) have been seen as an important target for the control of different diseases, such as leishmaniasis. Thus, in this article we present the principal strategies to efficiently induce activation of DCs in the context of leishmaniasis.

## Dendritic cells as the Main Target of Vaccines Against *Leishmania*

The major task for a vaccine is to correctly induce the immune system to develop a protective response against one specific pathogen. In the case of leishmaniasis, this protection comes from Th1 CD4^+^ T cells producing IFN-γ, TNF, and IL-12, which have been associated with disease control, macrophage activation, and elimination of parasites (5). However, to initiate this response, antigen presenting cells (APCs), present in different tissues of the body, must be activated to induce a proper response to eliminate or control the parasite. DCs are highly specialized APCs of the immune system capable of priming naïve T cells, and mounting a T-cell response upon pathogen entry in the body. Distinct subsets of DCs are associated with lineages and receptor expression patterns (6), and they develop from hematopoietic stem cells stimulated with fms-like tyrosine kinase 3 ligand (Flt3L) or with granulocyte/macrophage colony-stimulating factor (GM-CSF) (7). The majority of DCs develop from myeloid precursors, whereas plasmacytoid DC (pDC) develops from lymphoid precursors and shares many features with B cells (8). To aid in their function, DCs express different toll-like receptors (TLR), which bind to common molecules associated with pathogens, and have been target for the development of new vaccine adjuvants. DCs express a large variety of receptors involved with uptake of molecules and pathogens, such as DC-SIGN (CD209) and DEC205 (CD205) (9). Immature DCs have a high endocytic capacity, which leads to pathogen or pathogen’s antigens degradation, processing, and finally loading of major histocompatibility complex (MHC) molecules. Later, mature DCs lose their high capacity of endocytosis, and change efforts to up-regulate the expression of several receptors for cytokines, MHC, adhesion, and co-stimulatory molecules, such as CD80, CD86, and CD40. It is estimated that each mature DC expresses around 10^6^–10^7^ MHC Class II and 10^5^ MHC Class I molecules, and “fix” a repertoire of peptides bounded onto MHC Class II to present to T cells (10). Eventually, DCs acquire capacity to activate a specific T-cell response against the pathogen that induced its activation (11). The last years were marked by an increase in the knowledge on the role and function of DCs in the immune system, and thus, the emergence of potential applications based on its manipulation. Starting on the field of cancer, which finally led to the US FDA approval of a DC-based vaccine against prostate cancer (Sipuleucel-T, Provenge^®^) (12), applications were quickly transferred to the field of infectious diseases with very encouraging clinical results against HIV (13). Although prospective results for infectious diseases were achieved, there is no DC vaccine or therapy for any infectious diseases that are currently available or in the pipeline (14, 15). Most of the results were gathered by *ex vivo* manipulation of DCs, and *in vitro* assays by loading it with desired antigens.

## *Ex vivo* Assays with DCs

Different researches have shown interesting results with murine models vaccinated with DCs *ex vivo* loaded with leishmania lysate antigen (14, 16). These results were encouraging since they showed that animals presented low levels of parasite burden and high levels of protective cytokines from the Th1 profile after vaccination. DCs engineered to overexpress IL-12 were successfully used for both vaccination and immunotherapy of murine models with infections already established (17). Interestingly, it was also reported that different subsets of DCs may induce different responses upon activation. Vaccination of BALB/c mice with pDCs pulsed with *L. major* complete antigen showed that they were protected against subsequent infection (18). In parallel, the use of DCs pulsed with *L. donovani*-soluble antigen combined with chemotherapy with pentavalent antimonials was able to eradicate parasites from infected mice (19). Curiously, regarding viability of DCs present in the vaccine, Schnitzer et al. (20) have reported that fragments of DCs or exosomes derived from DCs that had been previously exposed to parasite lysate of *L. major*, conferred protection in susceptible mice. Although modulation of DCs activity is highly dependent on the specie of *Leishmania* causing the infection, some of these studies were performed using *L. major* that is responsible for cases of CL. Thereby, there is an outstanding importance of studies dedicated to evaluate the action of molecular defined antigens of *Leishmania* spp. in the activity of DCs as a target for vaccines. Furthermore, many antigens from the *Leishmania* spp. proteome have unknown function and could be key-molecules to induce protective immunity. The importance of using defined antigens is so expressive that DCs pulsed with peptide (154–169aa) from gp63 induced a Th1 protection in BALB/c mice infected with *L. major*, while stimulation with a second peptide (467–482aa) resulted in a Th2 shift and disease exacerbation (21). In this sense, computational immunology has been constantly increasing its value, and now diverse *in silico* approaches are available for identification of potential epitopes and antigens for vaccines, since experimental methods are difficult and time-consuming (22). In addition, the DNA sequencing techniques are getting cheaper, therefore, many parasite genome strains can be sequenced and their predicted proteomes can be assessed regarding their variability, an important feature for antigen candidates for vaccine development. Thus, sequence- and structure-based methods investigating the binding affinity of peptides to the MHC molecules Class I and II, and other parameters such as sequence diversity may aid in the search of new antigens (23). Taking advantages of these methods, Agallou et al. (24) have recently reported the construction of a multi-epitope peptide vaccine against leishmaniasis by analyzing four known proteins from *L. infantum*. In this last study, some of the proteins (histone H1 and KMP-11) have been previously tested in DCs vaccines against *L. infantum* (25, 26). Different studies for vaccines against *Leishmania* spp. have used defined molecules for classical immunization, without considering aspects of DCs activation, and maintenance of a Th1 response by memory cells. DCs have been implicated with the induction of a Th1 immune response through the production of IL-12, as seen in C57BL/6 and C3H resistant mice strains. On the other hand, BALB/c susceptible mice are more prone to produce a Th2 immune response with the presence of IL-4, IL-5, and IL-13; IL-4^−/−^ BALB/c mice were capable to partially control the infection with some strains of *L. major* (27). It has been shown that IL-4 during the early phase of DC activation induces a potent Th1 response induced by IL-12 (28); and in the context of vaccination with DCs, it was reported that IL-4 might be an important adjuvant, since IL-4Rα signaling is key to promote a Th1 immune response (14). Studies have enrolled IL-10 with disease progression by means of suppression of the anti-leishmanial immune response in humans and mice (29–31). Recently, Schwarz et al. (16) have shown that T cell are the main source of IL-10 in early infection, however, BALB/c mice vaccinated with fragmented DCs that had been pulsed with *L. major* lysate and CpG oligodeoxynucleotides (CpG-ODN) were able to suppress IL-10 favoring the control of infection. One of the main concerns in the vaccine development is the use of adjuvants, in this regard, DC approaches in leishmaniasis studies have used, mainly, CpG-ODN, which is a TLR9 ligand (32). The effect of CpG-ODN is to induce DC activation and maturation, enhancing humoral responses, with Th1 indicator IgG2a antibodies, and activity of cytotoxic T lymphocytes (CTL) (33, 34). It has been shown that CpG-ODN induce the production of CXCL10, a chemokine with anti-leishmanial properties, regulation of parasitic load, and CD4^+^CD25^+^ regulatory T (Treg) cells in mice infected with *L. donovani* (35, 36). Some issues may concern this approach of *ex vivo* activation, such as the type of antigen that is used, the method itself, which is time-consuming and labor, and also the high risk of infection.

## Overall Advantages of *In vivo* Activation of DCs

Toward a more rational and feasible DC-based vaccine against different pathogens, groups of research have endeavored to *in vivo* manipulate DCs, and thus, trying to understand the ways of induction a specific T cell response. In a very interesting work, Bonifaz et al. (37) have demonstrated that it is possible to manipulate DCs *in vivo* by directing antigen-conjugated antibodies against uptake receptors (DEC205). Other works followed the same strategy and achieved promising results for different pathogens, such as HIV, and also induction of immunity in distinct sites that are challenges for classical route immunization, such as mucosal sites (38, 39). For *L. major*, it was shown that *in vivo* targeting of DEC205 receptor of DCs with different antigen (LACK, LeIF, LmSTI1a)-conjugated antibodies can elicit a protective immune response with INF-γ and TNF-α in different mice strains (40). Therefore, based on these results collected during the last years, other uptake receptors could be a target to proper induce DCs maturation and activation. Nevertheless, as well as for other approaches, for a vaccine against *Leishmania* is not different, as there is a big gap in translating the research from murine models to humans due to environmental and genetic differences (41). Since murine models are difficult and do not always reproduce *Leishmania* infection, it might be interesting to test some of these approaches with cells from individuals affected or living in endemic regions, like recently described for other types of vaccine (42). Another ongoing challenge to be considered is the number of DCs present *in vivo*. DCs *in vivo* are rarer than other leukocyte populations. To overcome this issue, it has been shown that using Flt3L *in vivo* through gene transfection, one can enhance the number of DCs *in vivo* (43). In this context, it is worth mentioning the importance of works designed for VL, due to its high mortality level. However, in terms of number of cases and morbidity, CL and MCL are much more expressive than VL, especially in developing nations, such as Brazil.

## Conclusion and Perspectives

The results of DCs-based vaccines against leishmaniasis are very encouraging; demonstrating that either *ex vivo* or *in vivo* target of DCs can elicit an effective immune response to combat *Leishmania* spp. However, a number of biological and methodological challenges should be overcome prior to the development of a DCs-based vaccine. It is possible that moving researches forward to animal, and then, clinical studies will better point out if the target of DCs is safe and effective for an anti-*Leishmania* spp. vaccine.

## Conflict of Interest Statement

The authors declare that the research was conducted in the absence of any commercial or financial relationships that could be construed as a potential conflict of interest.
